# An Intelligent Logic-Based Mold Breakout Prediction System Algorithm for the Continuous Casting Process of Steel: A Novel Study

**DOI:** 10.3390/mi13122148

**Published:** 2022-12-05

**Authors:** Md Obaidullah Ansari, Joyjeet Ghose, Somnath Chattopadhyaya, Debasree Ghosh, Shubham Sharma, Prashant Sharma, Abhinav Kumar, Changhe Li, Rajesh Singh, Sayed M. Eldin

**Affiliations:** 1 Indian Institute of Technology, Dhanbad 826004, India; 2 Department of Production & Industrial Engineering, Birla Institute of Technology Mesra, Ranchi 835215, India; 3 Department of Chemical Engineering, Birla Institute of Technology Mesra, Ranchi 835215, India; 4 Mechanical Engineering Department, University Center for Research & Development, Chandigarh University, Mohali 140413, India; 5 School of Mechanical and Automotive Engineering, Qingdao University of Technology, Qingdao 266520, China; 6 Department of Civil Engineering, GLA University, Mathura 281406, India; 7 Department of Nuclear and Renewable Energy, Ural Federal University Named After the First President of Russia, Boris Yeltsin, 19 Mira Street, 620002 Ekaterinburg, Russia; 8 Uttaranchal Institute of Technology, Uttaranchal University, Dehradun 248007, India; 9 Department of Project Management, Universidad Internacional Iberoamericana, Campeche 24560, Mexico; 10 Centre for Research, Faculty of Engineering, Future University in Egypt, New Cairo 11835, Egypt

**Keywords:** continuous casting machine, logic-judgment-based model, sticker breakouts

## Abstract

Mold breakout is one of the significant problems in a continuous casting machine (caster). It represents one of the key areas within the steel production facilities of a steel plant. A breakout event on a caster will always cause safety hazards, high repair costs, loss of production, and shutdown of the caster for a short while. In this paper, a logic-judgment-based mold breakout prediction system has been developed for a continuous casting machine. This system developed new algorithms to detect the different sticker behaviors. With more algorithms running, each algorithm is more specialized in the other behaviors of stickers. This new logic-based breakout prediction system (BOPS) not only detects sticker breakouts but also detects breakouts that takes place due to variations in casting speed, mold level fluctuation, and taper/mold problems. This system also finds the exact location of the breakout in the mold and reduces the number of false alarms. The task of the system is to recognize a sticker and prevent a breakout. Moreover, the breakout prediction system uses an online thermal map of the mold for process visualization and assisting breakout prediction. This is done by alerting the operating staff or automatically reducing the cast speed according to the location of alarmed thermocouples, the type of steel, the tundish temperature, and the size of the cold slab width. By applying the proposed model in an actual steel plant, field application results show that it could timely detect all 13 breakouts with a detection ratio of 100%, and the frequency of false alarms was less than 0.056% times/heat. It has the additional advantage of not needing a lot of learning data, as most neural networks do. Thus, this new logical BOPS system should not only detect the sticker breakouts but also detect breakouts taking place due to variations in casting speed and mold level fluctuation.

## 1. Introduction

Industrially steel consumption is still huge. Therefore, very high steel production is a prerequisite for any steel plant. Modern integrated steel plants are designed for high production output with a wide range of steel products. Continuous casting represents one of the key areas within the steel-producing facilities of a steel plant. Continuous casting of steel is a process in which liquid steel is continuously solidified into a strand of metal. Depending on the dimensions of the strand, these semifinished products are called slabs, blooms, or billets. Today, more than 90% of steel is produced using continuous casting [1,2,3].

However, the continuous casting process has numerous problems which need to be overcome for producing cost-effective, quality steel. One such problem is breakouts, which lead to a huge loss of production as well as revenue. A breakout event on a caster often causes safety hazards, high repair costs, loss of production, and shutdown of the caster for a short while [4]. Moreover, breakout rates have increased with the recent trend of increasing casting speeds to enhance production rates [5,6]. The most common problem leading to breakouts is a so-called “sticker” inside the mold [7]. It is characterized by the sticking of the strand shell to the mold copper plate due to a lubrication fault.

**Figure 1:**
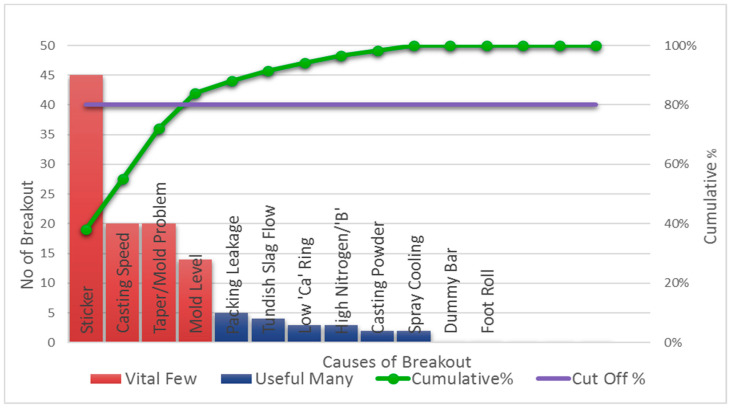
Pareto Analysis of reasons for breakouts at BSPL.

The mold breakout data from the operational logbook of Bokaro Steel Plant (BSPL) indicates that breakouts can occur for many reasons. The Pareto analysis chart in Figure 1 shows that approximately 84% of mold breakouts take place due to sticker formations, casting speed, taper/mold problems, and mold levels. The literature also indicates that more than 80% of mold breakouts occur due to stickers [4,8]. Approximately 31.15 million INR/year of liquid steel is lost due to stickers, casting speed, taper/mold problems, and mold-level breakouts, as shown in Table 1 [9].

Deviations from optimum casting conditions must be detected as soon as possible. The task of the breakout prediction system (BOPS) is to recognize a sticker and prevent a breakout. This is done by alerting the operating staff or automatically reducing the cast speed [10].

## 2. A Review of the Literature

Over the years, many breakout prediction technologies have been developed. Such BOPSs can be divided into two categories: artificial-intelligence-based methods and logic-judgment-based methods. Ansari, Md Obaidullah et al. developed a back-propagation neural network to predict the presence of a primary crack that might lead to the breakout of liquid steel [11]. Liu, Yu et al. constructed a genetic algorithm of a Levenberg–Marquardt-based back propagation neural network model to detect mold breakouts with a higher accuracy rate (83.3%) and lower false alarm rate (0.05%) [12]. Cheng, Ji et al. presented a compound sticking breakout prediction model including two kinds of modules: the time-sequence module of a single thermocouple and the space module of a multithermocouple; testing results showed the quote rate and accuracy rate for sticker breakout prediction have both achieved 100% [13]. Tirian, Gelu-Ovidiu et al. presented an adaptive control system for continuous steel casting based on a neural network and fuzzy logic for crack prediction [14,15]. Wang, Yanyu et al. used computer vision technology to visualize the temperature of mold copper plates, extract the geometric and movement characteristics of the sticking region from time and space perspectives, and construct feature vectors to characterize the V-shaped sticker breakout region [16]. Duan, Haiyang et al. compared a logical judgment and artificial neural network; the method based on clustering did not need to modify forecast thresholds or parameters artificially, which overcame the limitation of model-dependence on human beings and demonstrated excellent adaptability and robustness for online abnormality prevention [17]. Bellomo, P et al. developed a neural network and utilized it for breakout monitoring [18]. Bouhouche, Salah developed a mathematical model using an advanced approach based on a neural network and applied it to the control and quality optimization in the main processes of steel production. This system improved the breakout prediction system and reduced the rate of false alarms generated by the conventional breakout detection system [19]. Zhang, Ben-Guo et al. presented a back propagation neural network based on the Levenberg–Marquardt algorithm and applied it to the breakout prediction system in the continuous casting process. The results showed that the accuracy rate of the model was 96.43%, and the quote rate was 100% [20]. Artificial-intelligence-based methods can predict breakouts using pattern recognition algorithms, such as artificial neural networks, support vector machines, k-mean clustering, quantum wavelet neural network, etc. Intelligent systems have good prediction accuracy, stronger adaptation, self-learning capabilities, fault tolerance, and robustness for solving actual nonlinear problems. However, an artificial-intelligence-based breakout prediction system is a “black box” model [21,22,23], lacks technological guidance, and excessively depends on learning data. This often leads to failure of the system.

He, Fei et al. presented a new system to predict and prevent the sticking type of breakout in slab continuous casting; it used a novel logic judgment model for sticker prediction [24]. Blažević, D., M. Ikonić, and T. Mikac are developing an appropriate algorithm for the prevention of molten steel sticking onto molds. The logic of such an algorithm is simple and manageable to ensure its practical implementation in a computer system via the usage of thermocouple sensors [25]. Langer, M. and Arzberger, M. are now employing a field-bus-based system to digitize data at the mold along with suitable algorithms, which enables the caster to be stopped within two seconds to heal the strand when potential breakout conditions are detected [26]. Itoyama, Seiji et al. developed a logic-based algorithm to predict and prevent sticker breakouts [27]. Emling, W.H. and S1 Dawson developed a logic-based breakout prediction system to predict liquid steel breakouts with a higher accuracy rate and lower false alarm rate [28]. Hewitt, P.N. et al. used mold thermal monitoring to detect sticker breakouts and later to develop casting powder practices and provide an online assessment of mold conditions, improving performance in the mold in terms of casting flux evaluation, detection of preferential flow, and detecting longitudinal cracks [29]. Normanton, A.S. et al. briefly outlined recent developments in mold thermal monitoring systems; the prime function of the systems is to detect and prevent sticker breakouts [30]. Watzinger, J. et al. installed a mold expert monitoring system to predict longitudinal surface cracks based on thermal and frictional behavior in the mold [31]. Yang, Jie et al presented the application and optimization of mold breakout technology to predict and prevent liquid steel breakouts by using different logic algorithms [32]. The logic-judgment-based method is based on qualitative and quantitative analysis of the process parameters, actual breakout data, and logic algorithms. Logic-judgment-based breakout predictions are widely used and play an important role in reducing sticker breakouts. The main problems in these systems are high false alarm rates and poor robustness due to the reading and reliability of thermocouples. These issues must be considered when designing logical algorithms.

When any BOPS raises a sticker alarm, reducing the casting speed is the primary method to recover the sticker and prevent a breakout. Such a reduction in casting speed can be achieved manually or automatically. Many researchers [6,33,34,35] reported that breakouts can be prevented by reducing casting speed. In actual production, breakouts still occur after alarms due to late alarms or improper control strategies of the casting speed.

The literature shows logic-judgment-based systems has several advantages over artificial-intelligence-based systems, such as being easy to implement and having no need for learning data [5,36,37,38]. However, such logic-judgment-based BOPSs needs to be carefully designed to overcome the problems of false alarms and lack of robustness.

In this work, a novel logic-judgment-based BOPS has been developed for a continuous casting system. In this BOPS, new algorithms have been implemented to detect different sticker behaviors. In this BOPS, several algorithms are running simultaneously, and each algorithm is specialized in different sticker behaviors. This also reduces the number of false alarms. This system can identify different grades of steel automatically based on distinct thermocouple behaviors. The algorithm’s parameters are set accordingly for the different solidification behaviors of steel grades. This system’s job is to recognize a sticker and prevent a breakout. In addition, the breakout prediction system makes use of an online heat map of the mold to aid process visualization and breakout prediction. This is accomplished by either notifying the operational team or automatically lowering the cast speed. It also has the advantage of not requiring as much learning data as neural networks and being simple to integrate into existing systems.

## 3. Process Description

### 3.1. Sticker Breakout Identification

**Figure 2:**
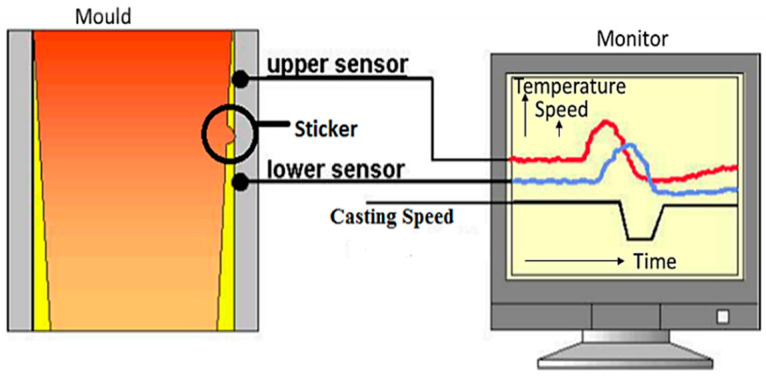
Temperature sensor measurements display.

The development of stickers in the caster can be easily identified by their characteristic temperature patterns in the copper mold. These temperatures are measured by the installed thermocouples in the upper part of the mold. The number and location of sensors depend on the geometry of the mold and the maximum casting speed. The BOPS is based on checking the temperature relations of sensor pairs. Such a pair consists of two thermocouples from a single column, as shown in Figure 2.

### 3.2. Sticker Development

**Figure 3:**
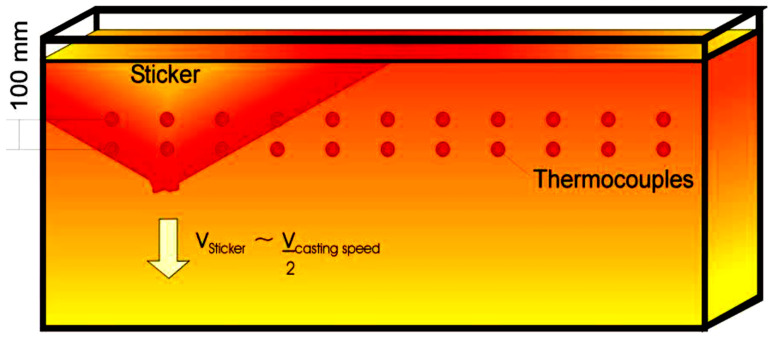
Sticker development in the mold.

A sticker is formed below the mold level where the strand shell is very thin, as shown in Figure 3. The lubrication film of the molten powder is insufficiently distributed in this region, and therefore, the shell is directly attached to the copper mold surface. Thus, an area of high friction emerges, and the steel shell below the sticker opens. The hole then deepens with each oscillation. The reason for sticking mainly traces back to bad lubrication of the casting powder, large mold level fluctuations, unskilled steel melting, and unsuitable taper adjustment [39,40,41]. Breakouts can also be caused by generally unstable casting conditions and operator actions such as rapid speed changes.

### 3.3. Sticker Detection Logic

**Figure 4:**
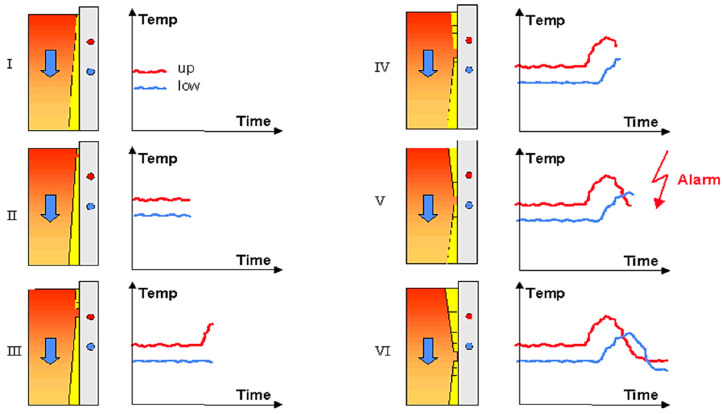
Sticker development temperature variation in sensors.

A typical temperature plot during the development of a sticker can be seen in Figure 4. During normal casting conditions, the average temperature of the upper thermocouple is higher than that of the lower one due to the growth of the strand shell (step I). When a sticker starts in the mold (step II), the upper thermocouple temperature rises because of the defect in the shell. The temperature of the lower thermocouple is not affected (step III). Later, depending on the casting speed, the sticker has passed the upper thermocouple and is reaching the lower one (step IV). The lower temperature is also rising while the upper temperature is decreasing again because it is no longer influenced by the sticker (step V). The upper temperature drops so much that it falls below the lower one, and the temperature difference can get negative (steps V + VI). This typical temperature pattern makes it possible to detect a sticker by monitoring and processing the temperature values of each sensor pair. If a sticker is detected, a breakout alarm is given, as shown in Figure 4.

When the sticker reaches the bottom of the mold, the molten steel leaks out from the mold. Such an accident is called a “breakout.” Breakouts lead to temporary shutdowns of slab caster, damage of machinery due to splashes of molten steel, capital loss, decreased productivity, and safety hazards.

### 3.4. The Copper Mold

**Figure 5:**
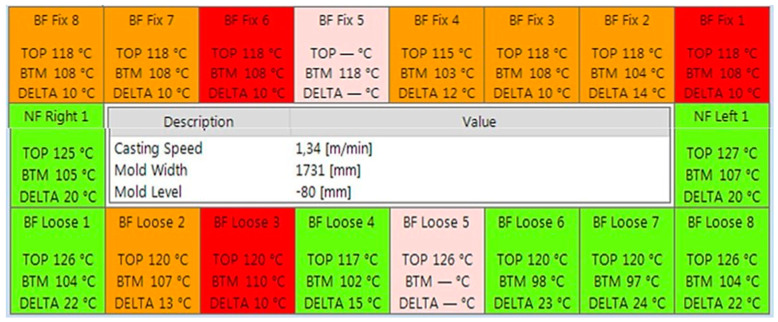
Arrangement of thermocouples installed in copper plates of the mold.

The mold is the caster’s heart, providing the necessary strand geometry and major heat removal during early growth of the strand shell. Convective heat transmission is efficient when primary cooling water is carried down the interior of the copper plate [42,43,44]. Width adjustment for the various section sizes is made by moving the narrow face assemblies of the mold. The narrow face assemblies can be shifted perpendicularly to the casting direction using an adjusting mechanism. The arrangement of thermocouples installed in copper plates of the mold is shown in Figure 5. Technical data and dimension detail of the mold are given in Table 2.

In the following table, thermocouples with the same horizontal position are said to form a thermocouple column, and thermocouples with the same vertical position are said to form a thermocouple row. Hence, the picture above shows two thermocouple rows and 36 columns.

## 4. Method: Research Methodology

### 4.1. Development of Logic-Judgment-Based BOP

Based on years of metallurgical experience, different autoadaptive algorithms have been developed to cover a wide area of shell disturbance that can cause or induce breakouts. Common to all algorithms is that the various limits and signal-filtering parameters are autoadaptive and automatically derived from the signal history. Here, a new logic judgment model was developed based on upper and lower thermocouple temperature gradient (temperature change rate), casting speed, mold level, monitoring of neighboring thermocouples’ temperatures, and sticker movement. This new logical BOPS should not only detect sticker breakouts but also detect breakouts taking place due to variations in casting speed and mold level fluctuation.

#### 4.1.1. Data Preprocessing

In a continuous casting shop, large fluctuations and malfunctions often occur in the temperature of thermocouples. This is due to the thermocouples being mechanically broken and open, the mold cooling system not working properly, faults in the analog-to-digital converter, etc. Data preprocessing was used to reduce unwanted data from the data set.

##### Thermocouple Temperature Rule

The maximum value of the thermocouple temperature (TC) as $\left({\mathrm{TC}}_{\max}=250\;℃\right)$ and the minimum value of thermocouple temperature as $\left({\mathrm{TC}}_{\min}=50\;℃\right)$ are predefined temperatures.

(1) $${\mathrm{TC}}_{\max}\geq \mathrm{TC}\geq {\mathrm{TC}}_{\min}$$

If TC satisfies Equation (1), then the thermocouple temperature value would be used in the calculation of the model.

Defective or faulty upper thermocouple temperatures will always show 300 °C, and defective lower thermocouple temperatures will always show as 0 °C. Faulty thermocouple readings negatively influence breakout prevention and should be replaced as soon as possible. The following includes the rules for exchanging a mold for faulty thermocouple readings.

##### Mold Replacement Is Not Mandatory

**Figure 6:**
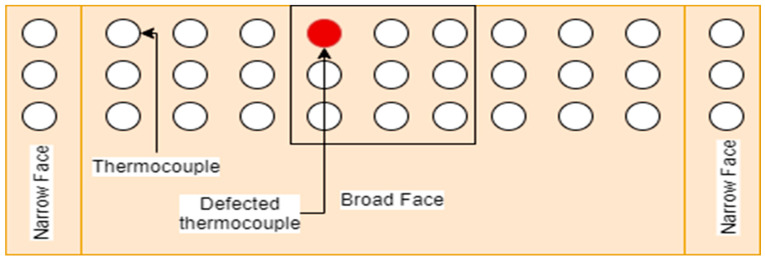
Mold replacement is not mandatory.

In any grid of 3 × 3 thermocouples, if only one thermocouple of any one column is not working, the mold can be used longer, as shown in Figure 6.

##### Recommended Mold Replacement

**Figure 7:**
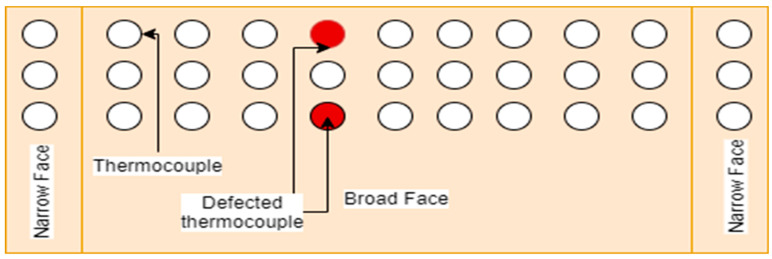
Mold replacement recommended.

In any grid of 3 × 3 thermocouples, if two or more thermocouples of any one column are not working, the mold should be exchanged, as shown in Figure 7.

### 4.2. Thermocouple Temperature Gradient Rule

The temperature gradient of each thermocouple can be calculated by Equations (2) and (3). When the rising temperature meets Equations (2) and (3), it is considered an abnormal temperature rise.

(2) $$\Theta =\frac{\mathrm{dTC}}{\mathrm{dt}}=\frac{\mathrm{TC}\left(\ldots ,j,{\;t}_{\mathrm{present}}\right)-\mathrm{TC}\left(\ldots ,j,t_{\mathrm{previous}}\right)}{t_{\mathrm{present}}-t_{\mathrm{previous}}}\;$$

(3) $$\theta_{\max}\geq \theta \geq \theta_{\min}\;\;\mathrm{and}\;t_{\max}\geq t\geq t_{\min}$$

where, θ is the rate of change of temperature of thermocouple, θ≈0 ℃/s for normal condition, t is the time, $\mathrm{TC}\left(\ldots ,j,t_{\mathrm{present}}\right)-\mathrm{TC}\left(\ldots ,j,t_{\mathrm{previous}}\right)$ are thermocouple temperature of ith row and jth column present and previous, respectively, $\theta_{\max}=2.20\;℃/s\;\mathrm{and}\;\theta_{\min}=0.18\;℃/s$ are the predefined maximum and the minimum rate of change in temperature, respectively, $t_{\max}=25{\;s\;\mathrm{and}\;t}_{\min}=5\;s$ are the predefined maximum and minimum of the duration, respectively.

### 4.3. Casting Speed Rule

Casting speed (CS), ${\mathrm{CS}}_{\max}=0.70\;m/\min$, and ${\mathrm{CS}}_{\min}=1.50\;m/\min$ are predefined casting speeds.

(4) $${\mathrm{CS}}_{\max}\geq \mathrm{CS}\geq {\mathrm{CS}}_{\min}$$

If CS satisfies Equation (4), then the casting speed value is used in the calculation of the model.

### 4.4. Mold Level

Mold level (ML) is always greater than or equal to 20% of the predefined value. If the ML satisfies Equation (5), then the mold level value participates in the calculation of the model.

(5) ML≥20%

#### 4.4.1. Speed of Sticker in the Vertical Direction Rule

If $S_{s}$ is the speed of the sticker in the vertical direction, $C_{s}$ is casting speed, $D_{v}=100\;\mathrm{mm}$ is the predefine vertical distance, and $t_{v}$ is the time required to move the sticker in the vertical direction between two consecutive thermocouples, respectively, and α is the predefine ratio of the speed of the sticker to casting speed. $\alpha_{\min}=0.38{\;\mathrm{and}\;\alpha }_{\max}=1.50$. The following equations must be satisfied:

(6) Cs≥Ss=Dvtv

(7) $$\alpha \geq \frac{S_{s}}{C_{s}}$$

(8) $$\alpha_{\max}\geq \alpha \geq \alpha_{\min}$$

#### 4.4.2. Breakout Detection Algorithms

**Figure 8:**
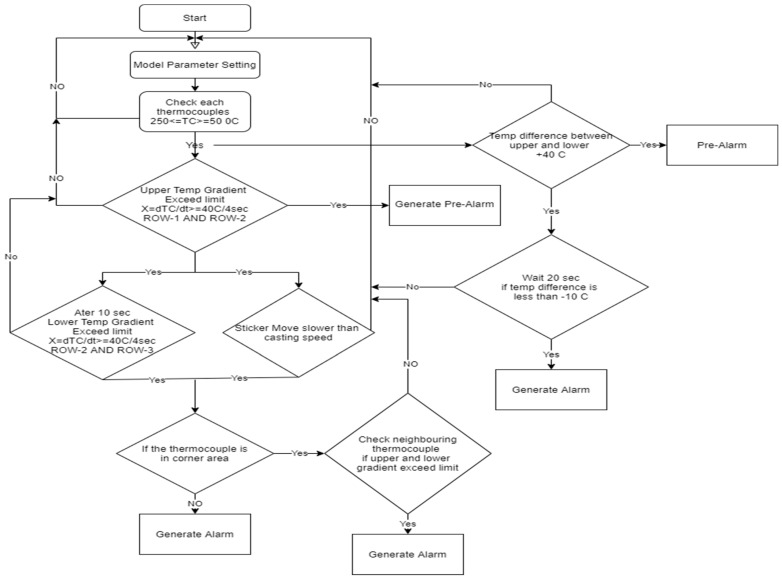
New logic-based model for sticker breakout prediction.

The sticker detection algorithms always need pairs of thermocouple readings to work on. For instance, if the mold is equipped with three rows of thermocouples, one algorithm could use the pairs of row-1 and row-2 thermocouples, another one could use the pairs of row-2 and row-3 thermocouples, and yet another one could use the pairs of row-1 and row-3 thermocouples. The algorithms (“Temperature-Gradient-Algorithm”) and (“Temperature-Difference-Algorithm”) work on each thermocouple column around the mold (broad face fix, narrow face right, broad face loose, narrow face left) independently from the other thermocouple columns. The algorithm (“Extended-Temperature-Gradient-Algorithm“) also takes into account the next neighboring thermocouple column that is in a normal operational state. If at least one thermocouple of the two rows is disabled (automatically or manually by the operator), that thermocouple pair no longer participates in sticker detection. This part of the mold face is no longer protected by the sticker detection algorithms (“Temperature-Gradient-Algorithm”) and (“Temperature-Difference-Algorithm”). The algorithm (“Extended-Temperature-Gradient-Algorithm”) still has a certain low probability to detect a sticker formation in this area by taking into account the next neighboring thermocouple column. Details of sticker detection algorithms are given in Table 3. A thermocouple with apparently “wrong” or “weird” temperature readings should be disabled because such temperatures can cause the sticker detection algorithms to generate false alarms as illustrated in Figure 8.

With this mold breakout prediction system for all sticker detection algorithms, all three rows with all combinations of two vertical thermocouples will be used. This helps to detect stickers that even show no significant temperature increase in the first row. Further, it is possible to also detect stickers in a column where the information of one thermocouple is missing (e.g., because of a faulty signal) [44,45,46]. The biggest advantage is that the system is still using the combination of the remaining pair of thermocouples. So, it keeps a column of thermocouples working, even if one of the three thermocouples is defective [47,48,49,50,51,52,53,54,55,56,57,58,59]. Only if two thermocouples in one column are not working properly, no breakout prevention can be done. Nevertheless, it is recommended to change the thermocouples in case of malfunction as soon as possible.

There are special, unstable casting conditions when a breakout alarm of the BOPS does not stop the caster. Those unstable casting conditions are:

- Cast length after the start of the casting is less than 2 m.
- Mold level changes by more than 20 mm within the last 60 s.
- Casting speed is <0.2 or >10 m/min within the last 60 s or casting speed change is larger than 7.2 m/min² within the last 60 s.

## 5. Results and Discussions

The primary function of this work is to recognize a sticker and prevent breakout. This is done by giving information to the operating staff or automatically reducing the casting speed. This new “logical-based breakout prediction system (BOPS)” not only detects the “sticker-type breakout” but also detects the breakout that takes place due to variation in “casting speed”, “mold level fluctuation”, and “taper/mold problem”. Additionally, the “BOPS” employs an online “thermal map of the mold” to aid with “breakout prediction”, and “process visualization”. The field application results reveal that the designed BOPS could identify all 13 breakouts in real-time with only three false alarms.

### 5.1. Simulated Experiment

**Figure 9:**
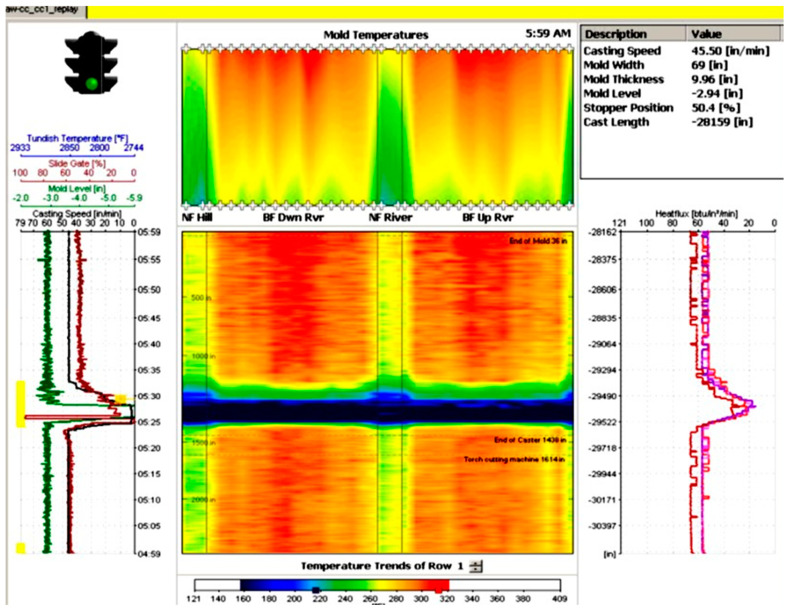
Entire conditions of the casting process inside the mold.

The process monitor is the main monitor of the mold expert system which represents the most important information and gives an overview of the entire condition of the casting process inside the mold. It provides a survey of the mold thermal condition, tundish temperature, slide gate or stopper position, mold level, casting speed, mold heat flux, and the alarm status as exhibited in Figure 9. The thermal condition of the mold is based on the measured values of the thermocouples. The task of the mold expert system is to recognize a sticker and prevent a breakout. This is done by alarming the operating staff or automatically reducing the cast speed.

The conditions under which the current casting situation is considered unstable can be configured by a user with configuration rights via the mold expert configuration form. Table 4 show the conditions which would prevent a breakout alarm.

To validate the aforementioned logic principles and choose the ideal model parameters, the simulated trials of new BOPS were conducted based on past breakout data that was obtained from the BSP logbook. The ideal model parameters were established after repeated fine-tuning through alarm analysis, as illustrated in Table 5. Two scenarios—stable casting conditions and unstable casting conditions—were used to conduct simulated trials with the novel BOPS. Additionally, the BOPS employs an online thermal map of the mold to aid with breakout prediction and process visualization [50,51,52].

#### 5.1.1. Alarms during Stable Casting Conditions

**Figure 10:**
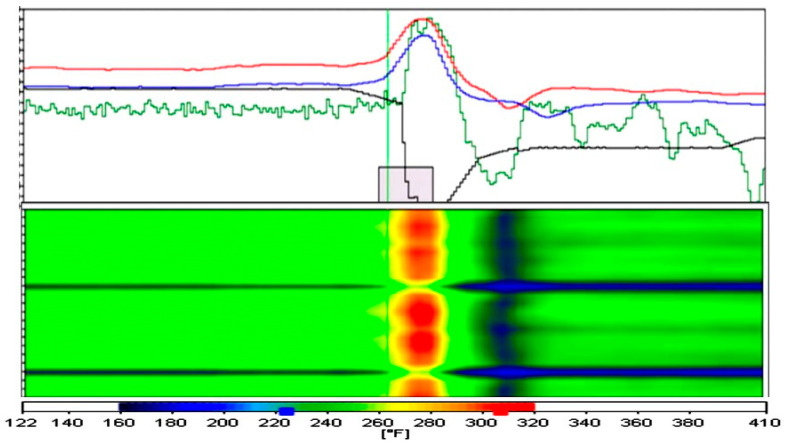
Alarms during stable casting conditions with temperature spectrum.

To begin, a specific example can be used to explain the process analysis of the new logical-based model in detail. This is a typical sticker breakout incident at bff1(1) and bff2(2) during steady casting circumstances, as seen in Figure 10. The gray bar at the bottom-middle of the graph indicates that there is a sticker breakout alarm, and the corresponding mold thermal condition also confirms that there is a sticker breakout at 22:38:11, as shown in Figure 10. Following the alarm, a breakout was averted within 60 seconds by slowing down the casting pace, allowing ample time to heal the sticker and prevent the breakout [53,54,55]. As demonstrated in Figure 10, there was a mapping between color and temperature.

#### 5.1.2. Alarms during Unstable Casting Conditions

During unstable casting conditions, breakout alarms are generated in three different situations, which are as follows:

- Breakout due to start cast during unstable casting condition
- Breakout due to casting speed change during unstable casting condition
- Breakout due to mold level change during unstable casting condition

### 5.2. Breakout during Start Cast

**Figure 11:**
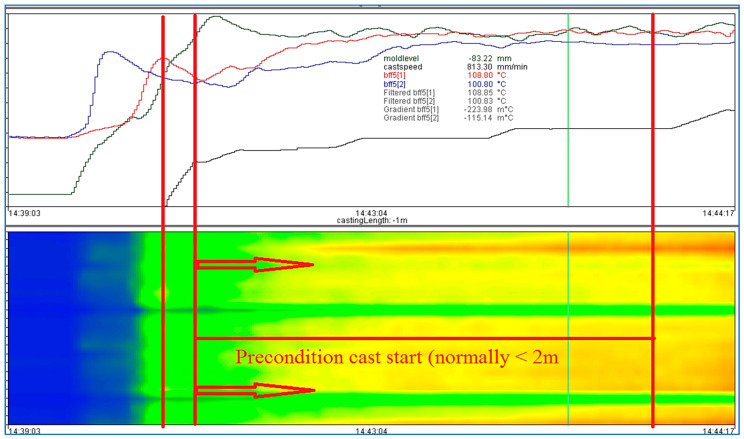
Start cast alarm during unstable casting condition.

Getting a steady operation going after startup and then sustaining stability are two of the biggest operational problems with the continuous casting process [36,56,59]. A steel dummy bar is used to seal the mold bottom before casting to stop molten steel from leaking out of the mold. While the system is starting up, a breakout might happen, sometimes referred to as a “start cast breakout” [37,58,59]. A cast start scenario, a high degree of casting speed change, or an unstable casting state are all indicators of this in the mold expert system. Alarms that occur during a cast start condition often do not cause the casting pace to slow down, only warnings will be displayed. Nevertheless, there are sometimes critical casting situations at cast start which can lead to a breakout. To prevent such breakouts, a special algorithm has been developed which is called the “cast start algorithm”. The reason for a breakout at cast start can be a sticker, but other reasons exist as well. However, there has to be an untypical temperature trend, such as a sticker-temperature trend, to detect the critical situation and reduce the casting speed automatically. During the process of filling the mold, there are very high and unstable temperature fluctuations. This casting part cannot be used for analysis because it would lead to too many false alarms. For this reason, a “Start Point” at which the cast start algorithm starts with its calculations has to be defined. This point is reached if a defined casting speed is reached. The algorithm will calculate until the precondition “cast start” is fulfilled, i.e., until a defined distance is cast after the cast speed crossed a defined casting speed (creep speed) upwards. Now, take an example of a start cast breakout to demonstrate the process analysis of the new logical-based model in detail, and find out whether the new model can generate the alarm or not during start cast breakout. This example is a sticker breakout incident at bff5(1) and bff5(2) during unstable casting conditions, which are shown in Figure 11.

#### 5.2.1. Breakout during Casting Speed Change

Casting speed breakout occurs when the shell in the mold becomes thinner and slag consumption reduces, increasing the chance of a sticker between the shell and the mold. Table 6 illustrates the number of stickers produced at various casting speeds. The stickers occuring at casting speeds of 0.8 and 0.9 m/min and should not be taken into account. The frequency of breakouts increases considerably at casting speeds of 1.0 to 1.2 m/min [38,60,61].

**Figure 12:**
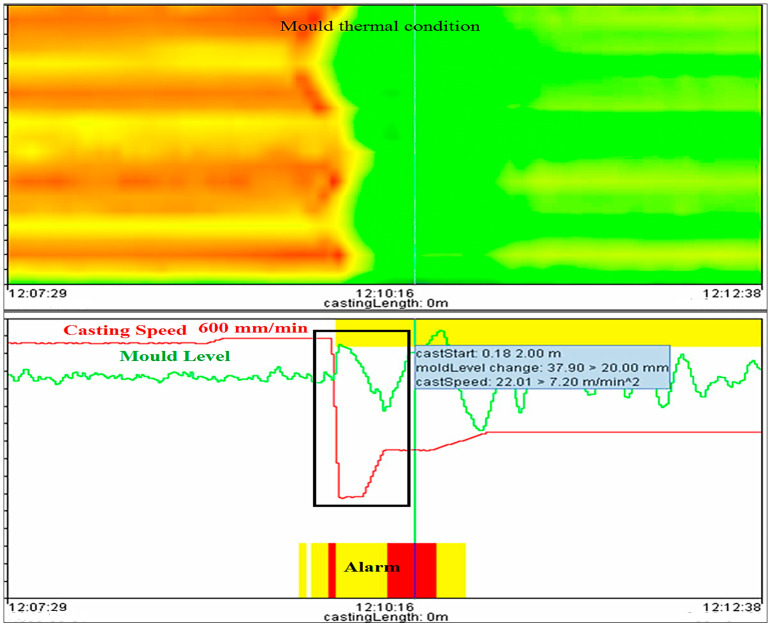
Breakout alarm due to variation in casting speed during unstable casting condition.

Now, take an example of a casting speed breakout to demonstrate the process analysis of the new logical-based model in detail, and find out whether the new model can generate the alarm or not during casting speed breakout. The yellow and red bar in the middle of the graph, as shown in Figure 12, indicates that there is a breakout alarm due to high casting speed (600 mm/min), and the crossponding mold thermal condition also confirms the breakout alarm. The mold thermal condition clearly shows that there is an unstable casting condition from 12:07:29 to 12:10:16; the shell in the mold grows thinner due to high casting speed. After the breakout alarm (12:10:16), the casting speed was reduced so that the liquid steel gets enough time and forms a thick shell within the mold and then maintains stability continuously.

#### 5.2.2. Breakout during Mold Level Fluctuation

Mold level fluctuation refers to transient fluctuations of the top free surface of the mold. Sudden mold level variations are connected with surface flaws in the final product, but when these fluctuations are particularly substantial, they cause mold level breakouts [39]. Table 6 [38] shows the relationship between the level fluctuation before sticking and the number of stickers. Table 7 clearly shows that the quantity of stickers rises as mold level drops.

**Figure 13:**
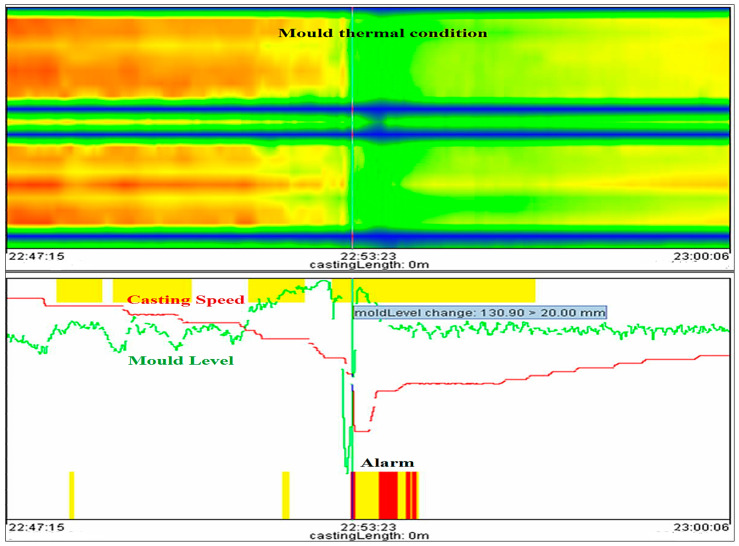
Breakout alarm due to variation in mold level during unstable casting condition.

Now, take an example of a mold level breakout to demonstrate the process analysis of the new logical-based model in detail, and find out whether the new model can generate the alarm or not during mold level breakout. The yellow and red bar at the middle of the graph, as shown in Figure 13, indicates that there is a breakout alarm due to a sudden fluctuation in mold level, and the crossponding mold thermal condition also confirms the breakout alarm. The mold thermal condition clearly shows that there is an unstable casting condition due to fluctuation of the mold level from 22:47:15 to 22:53:23; the shell in the mold becomes thinner due to the low mold level and does not get enough time to form a thicker shell. After the breakout alarm (22:53:23), the mold level increases and the casting speed is decreased so that the liquid steel gets enough time and forms a thick shell within the mold and then maintains stability continuously, as shown in Figure 13.

A total of 45 samples of sticker breakouts have been taken to find out whether the new model can detect the breakout or not. Simulation results showed that the new model could timely detect all 45 sticker breakouts without generating any false alarms. At the same time, the new BOPS model also detected the breakout that took place due to a change in casting speed. The simulation shows that all 20 breakouts can be readily detected without causing any false alarms. A total of 20 samples of casting speed breakout have been taken. The simulation results demonstrate that the novel technique detects all breakouts in real-time while minimizing the frequency of false warnings in 14 examples of mold level breakouts that were also obtained from previous BSP data. Another important component of continuous steel slab casting is the taper or mold [40,41,42,43,44]. Breakouts occur 20 times due to poor mold taper. A properly tapered mold accommodates for solidifying liquid steel shrinkage to ensure proper contact and heat transmission between the mold wall and semisolidified steel slab. An incorrectly tapered mold creates an air gap between the mold wall and the partially hardened steel slab, resulting in a breakout. As a result, before beginning the casting process, it is critical to appropriately taper the mold [62,63].

#### 5.2.3. Breakout Prevention Modes

**Figure 14:**
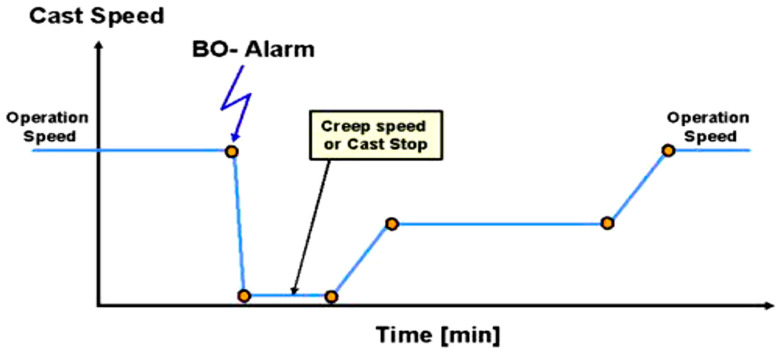
Breakout prevention.

After a sticker detection alarm, a breakout is prevented by stopping the caster or reducing the casting speed so that the defected shell will be healed. There are two different methods to react to an alarm event. First, (manual mode) a breakout alarm is sent to the operator who then reduces the casting speed or stops the caster manually. After a while, the operator increases the casting speed to the operating level. Second, (automatic mode) a breakout alarm stops the caster automatically and, after a while, the operator has to speed up the casting machine, as shown in Figure 14. An automatic increase of the casting speed is not advisable because the operator should have full control over the machine when a sticker occurs to inspect the situation in the mold and to increase the casting speed when the situation allows doing so. In automatic mode, after a sticker detection alarm, casting speed is reduced depending on the type of steel, tundish temperature, and size of cold slab width, as shown in Table 8.

### 5.3. Field Test

From April 2021 to September 2021, a new breakout prediction system was fitted to the slab continuous casters of the Bokaro Steel Factory for field experiments to assess the model’s performance and accuracy. The data for field testing were gathered from April 2021 to September 2021 with an average of 29.11 heat each day for a total of 5299.02 casting heat over six months. Table 9 shows that there were 13 breakouts over this time. The field application results reveal that the designed BOPS could identify all 13 breakouts in real-time with only three false alarms.

The detection ratio of the new model for breakouts was 100%, the frequency of false alarms was 0.05, and the prediction accuracy ratio was 100%. The detection ratio was calculated as follows: number of true alarms/(number of missed alarms + number of true alarms) for a breakout; the number of false alarms/total number of heats; and number of true alarms. Similar findings have been reported in earlier research, as indicated in Table 10, for analyzing false alarm time, multiple false alarms, and breakout detection ratio (%) as well as the breakout prediction accuracy ratio (%). Therefore, when compared to the data of other studies, our findings showed to be more trustworthy and significant in stopping any outbreak in the steel sector.

As a result, this methodology resulted in enhanced “product quality,” mitigating wastes/defects during the “production process,” and improving “productivity for steel slabs” while reducing “substantial capital loss” during the “continuous casting machine process” [61,62,63]. In light of that, the presented “hybrid algorithm” for “breakout prediction,” using a novel logic-judgment-based breakout prediction system algorithm with autoadaptive algorithms appears to be a more sustainable, viable, efficient, and cost-effective approach and perhaps is applicable to more advanced automated steel-manufacturing plant mills to accomplish the goals of Industry 4.0 [63,64,65].

## 6. Conclusions

Continuous casting machines which solve sticker breakouts are one of the major sections inside a steel plant’s steel manufacturing facilities. Furthermore, with the recent trend toward faster casting speeds, the breakout rate rises. In this study, a novel breakout prediction method has been developed not only to detect sticker breakouts but also to detect breakouts that take place due to variations in casting speed, mold level fluctuation, and taper/mold problems. Sticker breakouts were created by examining the features of the mold temperature change of each thermocouple and the recovery behavior of the sticker breakout. This method is based on qualitative and quantitative analyses of the process parameters, actual breakout data, and logic algorithms. It is also easy to implement and has no need for learning data. The following are the key findings of this investigation:

- The mold temperature change with time at each thermocouple, casting speed, mold level, tundish temperature, and tundish sliding gate were all used in this model.
- This model has new algorithms for detecting diverse sticker behaviors. With multiple algorithms operating, each algorithm becomes increasingly specialized in the various sticker behaviors.
- To test the correctness and performance of the proposed model, historical data with a genuine breakout was used to replicate the offered methods. The simulation results demonstrate that every breakout caused by sticker, casting speed, mold level, and taper/mold could be properly recognized while keeping the number of false alarms minimal.
- Field application findings of the proposed model in an actual steel factory demonstrated that it could timely identify all 13 breakouts with a detection ratio of 100% and a false alarm frequency of less than 0.056% times/heat.
- Our future research work will focus on mold oscillation (mold friction), heat flux, mold level, etc. that will further improve the mold expert system and also reduce the frequency of false alarm.

## Figures and Tables

**Table 1:** Revenue loss due to breakouts.

Particular	Value
**Number of breakouts**	23.6 per year
**For each breakout (an average of 3 to 4 hours delay) = (4 × 23.6) =** 94.4 h/year	
**Average loss of liquid steel**	3 tons per breakout
**Total liquid steel loss**	3 × 23.6 = 70.80 tons per year
**Cost of one ton of liquid steel**	44,000 INR
**Loss of liquid steel per year**	31.15 million INR/year

**Table 2:** Technical data of mold.

Types of Design	Straight Plate Mold with Width Adjustment
Width adjustment range	950−1650 mm
Thickness adjustment range	200−250 mm
Length of the copper plate	900 mm
Coating of copper plates	Ni-coating, 0.5/1.5 mm

**Table 3:** Sticker detection algorithm details for new logic judgment model.

Algorithm	Function	
**Temperature-Gradient Algorithm**	Basic algorithm for shell ruptures (shell sticker) detection	
	PRALARM	Upper temperature gradient exceeds limit
	ALARM	Lower temperature gradient exceeds limit and sticker moves slower than casting speed
**Extended-Temperature Gradient Algorithm**	Similar to “Temperature Gradient Algorithm” but with tighter parameter settings and monitoring of neighboring thermocouples for additional sensitivity. Especially useful for detection of shell disturbances in the mold corner areas.	
	PREALARM 1	Upper temperature gradient exceeds limit
	PREALARM 2	Lower temperature gradient exceeds limit and sticker moves slower than casting speed
	ALARM	Pre-alarm is confirmed by neighboring thermocouple column
**Temperature-Difference Algorithm**	For detection of strand shell disturbances based on moving disturbance	
	PREALARM	Temperature difference between upper and lower temperature exceed limit
	ALARM	Temperature difference gets negative and sticker move slower than casting speed
**Extended-Temperature Difference Algorithm**	Similar to “Temperature Difference Alarm” but with tighter parameter setting and monitoring of neighboring thermocouples for additional sensitivity.	
	PREALARM	Sticker is detected in one column
	ALARM	Sticker is confirmed by neighbor column
**Extended-Temperature Falling Algorithm**	Developed for stickers that do not show the typical increase of temperature when the sticker passes the thermocouple.	
	PREALARM	Sticker is detected in one column
	ALARM	Sticker is confirmed by neighbor column

**Table 4:** Breakout precondition.

Cast Start	Mold Level	Casting Speed
Active	Active	Active
Length 2.0 m	20.0 mm	Range 0.20–599,994.00 m/min Change 7.20 m/min^2^
Creep Speed 0.21 m/min	Duration 60 s	Duration 60 s

**Table 5:** Basic parameters list of the model.

Parameters	H (Higher Limit)	L (Lower Limit)
Thermocouple temperature	250 °C	50 °C
Casting speed	0.70 m/min	1.50 m/min
Mold level (average −97 mm)	−79 mm	−112 mm
Speed setpoint	0.00 m/min	0.00 m/min
Faulty upper thermocouple’s temperature shows 300 °C		
Faulty lower thermocouple’s temperature shows 0 °C		

**Table 6:** Number of stickers at various casting speeds.

**Steel Slab Dimension**	Length 900 mm, thickness 200–225 mm, and width 2000 mm				
**Casting speed**	0.8 m/min	0.9 m/min	1.0 m/min	1.1 m/min	1.2 m/min
**Stickers’ times**	1	1	3	2	15
**Frequency of stickers**	-	-	0.00256 times/hear	0.00272 times/hear	0.00505 times/hear

**Table 7:** Relationship between level fluctuation before sticking and number of stickers.

Mold Level	Less than or Equal to 3 mm	Equal to 4 mm	Equal to 5 mm	Equal to 6 mm	Greater than or Equal to 7 mm	Total
**Frequency of stickers**	9 times	10 times	7 times	3 times	2 times	31

**Table 8:** Casing speed depends on the type of steel, tundish temperature, and size of cold slab width.

Type of Steel	Tundish Temperature °C	Cold Slab Width (Size) in mm, Thickness: 225 mm				
		1850−1651	1650−1451	1450−1251	1250−1051	1050−950
			Casting Speed [m/min] Maximum			
**Low Carbon Steel C ≤ 0.08%**	≤1550	1.1	1.2	1.3	1.4	1.5
	1550–1560	1.0	1.1	1.2	1.3	1.4
	>1560	0.8	0.9	0.9	1.0	1.1
**Medium Carbon** **Steel C ≥ 0.08 to 0.15% (per)**	≤1550	1.0	1.1	1.2	1.2	1.3
	1550–1560	0.9	1.0	1.1	1.1	1.2
	>1560	0.8	0.9	0.9	1.0	1.0
**Carbon ≥ 0.15% to 0.22%**	≤1540	1.1	1.2	1.3	1.3	1.4
	1540–1545	1.0	1.1	1.2	1.3	1.4
	>1545	0.9	1.0	1.1	1.1	1.2
**C= 0.30–0.35 %**	≤1530	--	--	--	--	1.2
	>1530	--	--	--	--	1.1
	>1535	--	--	--	--	1.0
**C = 0.35–0.40 %**	≤1520	--	--	--	1.2	1.2
	>1520	--	--	--	1.1	1.1
	>1525	--	--	--	1.0	1.0
**C = 0.06–0.10%**	≤1540	1.0	1.1	1.2	1.2	1.2
	>1540	0.9	0.9	1.0	1.0	1.1
**Low Carbon microalloy steel C ≤ 0.08%**	≤1555	1.0	1.1	1.2	1.3	1.4
	>1555	0.9	0.9	1.0	1.2	1.3
**Peritectic microalloy** **C ≥ 0.08 to < 0.15%**	≤1555	1.0	1.1	1.2	1.3	1.1
	>1555	0.9	1.0	1.1	1.2	1.3
	>1560	0.8	0.8	0.9	1.0	1.1
**High Silicon** **Si > 0.10 %**	≤1555	1.0	1.1	1.2	1.3	1.1
	>1555	0.9	1.0	1.1	1.2	1.3
	>1560	0.8	0.8	0.9	1.0	1.1

**Table 9:** Breakout in continuous casting shop (CCS) from 1 April 2021 to 31 September 2021.

Breakout S.No.	Date and Time	Heat Number	Strand Number	Slab Size (mm)	Heat of Sequence	Ladle Number	Steel Grade	Casting Speed (m/min)	Mold Level (%)
		T1 (°C)	T2 (°C)	T3 (°C)	T4 (°C)	T5 (°C)	T6 (°C)	T7 (°C)	T8 (°C)
01	11.05.2021 06:30:30	53,969	02	1045	1st	14	CR2B	0.77	0
		186	8	146	9	10	76	−5	6
02	31.05.2021 04:10:02	54,335	02	1090	1st	21	GR-II	0.90	0
		188	12	132	3	−2	95	2	2
03	09.09.2021 03:17:56	57,263	04	1045	5th	13	GR-II	1.22	60
		13	130	20	14	−11	60	22	10
04	27.09.2021 22:50:31	57803	4	1090	4th	9	CR	1.01	62
		15	6	15	10	21	46	4	−11
05	07.10.2021 06:17:59	58,132	1 & 2	1470/1320	8th	18	GR-II Patton	1.32	64
		178	32	178	14	−15	169	31	30
06	28.10.2021 23:17:38	58,898	2	1045	8th	23	CR2	1.09	54
		194	0	125	−12	−8	125	−3	−3
07	31.10.2021 06:30:30	58,974	4	1320	6th	17	GR-I	1.02	60
		4	10	−13	5	4	210	−12	5
08	09.09.2021 20:47:33	57,263	4	1045	5th	13	GR-II	0.78	0
		2	−1	1	0	−6	1	−13	7
09	27.09.2021 02:44:48	57,803	4	1090	4th	9	CR	0.50	1
		54	−14	55	−13	−17	63	−16	−18
10	07.10.2021	58,132	1 & 2	1470/1320	8th	18	GR-II Patton	0.38	0
		−38	−6	93	−5	−9	49	−4	−5
11	28.10.2021 12:49:60	58,898	2	1045	8th	23	CR2	1.08	39
		−1	−1	−5	−17	−15	208	−12	1
12	31.10.2021 20:55:03	58,974	4	1320	6th	17	GR-I	1.22	60
		19	133	21	10	−11	51	18	14
13	03.11.2021 13:51:14	59,060	3	1320	5^th^	25	GR-II	0.77	0
		189	8	146	9	10	79	−5	6

**Table 10:** Comparison of the new model’s results with the data of other researchers.

Authors	Breakout Detection Ratio	Breakout Prediction Accuracy Ratio	Frequency of False Alarm
**New model**	100%	100%	0.056
**Ansari, Md Obaidullah et al.** [46]	100%	100%	0.113
**Liu, Yu et al.** [47]	98.73%	98.7%	0.126
**He, Fei et al.** [21]	100%	78.26%	0.150
**He, Fei et al.** [5]	100%	82.60%	0.1365
**Tian, Yuanpeng, and Yu Liu** [48]	100%	95.8%	0.840

## Data Availability

No data were used to support this study.
